# Influence of “Glow Discharge Plasma” as an External Stimulus on the Self-Assembly, Morphology and Binding Affinity of Gold Nanoparticle-Streptavidin Conjugates

**DOI:** 10.3390/ijms13066534

**Published:** 2012-05-29

**Authors:** Wael Mamdouh, Yingzhi Li, Sherif M. Shawky, Hassan M. E. Azzazy, Chang-Jun Liu

**Affiliations:** 1 Department of Chemistry, School of Sciences and Engineering, The American University in Cairo (AUC), AUC Avenue, P.O. Box 74, New Cairo 11835, Egypt; E-Mails: sherifshawky@aucegypt.edu (S.M.S.); hazzazy@aucegypt.edu (H.M.E.A.); 2 Advanced Nanotechnology Center, School of Chemical Engineering and Technology, Tianjin University, Tianjin 300072, China; E-Mails: dreamlyz@tju.edu.cn (Y.L.); coronacj@tju.edu.cn (C.-J.L.); 3 Yousef Jameel Science & Technology Research Center, the American University in Cairo, AUC Avenue, P.O. Box 74, New Cairo 11835, Egypt

**Keywords:** self-assembly, gold nanoparticles, interface, glow discharge plasma, atomic force microscopy, molecular aggregates, external stimuli

## Abstract

In this study, we investigate the influence of glow discharge plasma (GDP) on the self-assembly, morphology and binding affinity of streptavidin coated gold nanoparticles (Au-NP-SV) and biotinylated antibody (bAb) adsorbed on a highly oriented pyrolytic graphite (HOPG) substrate. Atomic force microscope (AFM) was used to image the pre- and post-GDP treated samples. The analysis of the AFM images showed a considerable change in the aggregation and morphology of Au-NP-conjugates after treatment with GDP. To our knowledge, this is the first report on using GDP to enhance and speed-up the aggregation (sintering) of adsorbed NP biomolecular conjugates. These results show a promising route that could be generalized for other NPs and their conjugates. It can also be considered as an alternative and cheap aggregation method for controlling the binding affinity of biomolecular species on different surfaces with interesting applications.

## 1. Introduction

The ability to control the self-assembly of molecules and their adsorption onto a surface is of fundamental importance [1] in many different applications such as developing novel nano-coating materials, inhibiting the adsorption of bacteria on biomedical implants’ surfaces (*i.e.*, developing antifouling surfaces), food industry, *etc*. [2]. A wide variety of molecular self-assembled systems have been explored ranging from simple/complex organic molecules to complex biological systems. Surface morphology and chemistry are among the most important factors that affect binding processes between molecular species. If the surface chemistry (*i.e.*, functionality) is altered, this will accordingly affect the binding efficiency between the adsorbed molecular species and the substrate surface. In biomaterials, altering the surface chemistry and composition of the biomedical implant material will have a significant impact on its ability to prevent the adsorption of bacteria and any undesired biomolecular adsorbates [3,4]. Antifouling surfaces have been revealed for that reason where controlling their surface properties has become essential [5].

External manipulations that can affect the surface properties is thus of great importance. Many external factors have been used to steer the self-assembly of biomolecular building blocks into larger nanostructures. A number of different nanostructures have been reported as a result of the influence of a wide variety of external stimuli on the self-assembly process [6]. Examples of such external factors are: metal cations [7], temperature [8,9], concentration [10,11], incubation time [8], pH value [11], pressure [12] for gas phase, and media solvent [13], *etc*.

Among the numerous types of nanoparticles, metal nanoparticles, especially gold, have attracted considerable attention recently because of their many interesting properties and potential technological applications. Varieties in synthetic strategies of nanoscale gold particles assembled into an aggregate structure have been reported [14].

Recently, a new strategy to directly monitor synthetic nanoparticles, metal objects and their transformations under a variety of environmental conditions by chemical and/or photoreduction has been introduced [15]. The strategy employed represented a new approach in which the fate of nanomaterials can be directly measured after exposure to a range of environmental stimuli. Glover *et al.* tethered silver nanoparticles (Ag-NPs) to an amine-functionalized silicon substrate, subjected them to varying environmental conditions such as temperature and humidity, and found that at room temperature and in humid air or in water, “daughter” silver nanoparticles spontaneously formed over days to weeks around “parent” nanoparticles. This humidity-dependent formation of new particles was broadly observed for a variety of Ag-NPs and substrate surface coatings. All silver, whether nano- or macro-scale, generated Ag-NPs when exposed to humid air or water. It is assumed that air oxidizes the silver and the resulting ions dissolve and diffuse in an adsorbed water layer on the substrate. New, smaller particles form by chemical and/or photoreduction of coalesced ions. Silver ions were released into, confined by, and reduced within the adsorbed water layer, leading to profoundly different reactivity than in solution. Copper objects were also found to display similar reactivity to the Ag-NPs and form nanoparticles as well, suggesting that this phenomenon may be more general.

It is now known that the intrinsic properties of metal nanoparticles are mainly governed by their size, shape, composition, crystallinity, and structure. In principle, one could control any one of these parameters to fine-tune the properties of these nanoparticles. If such tiny particles are allowed to coalesce in a controlled fashion, their spectroscopic properties as well as other properties such as color can be systematically varied. Whereas attention is focused primarily on the assembly of macroscopic crystals and films of dense cluster matter, there is also interest in developing methods for assembling small controlled aggregates of nanoparticles [14].

Plasma has been used as a measure for surface modification. The argon ion (Ar^+^) in the argon (Ar) plasma could break the *sp^2^* C-C bonds, and produce carbon free radicals (−C) and induce some physical and chemical defects on top of highly oriented pyrolytic graphite (HOPG) surface [16,17]. These defects cannot only react with O_2_ or H_2_O in air, but also act as nucleation sites of the noble metal particles. In addition, the plasma can also modify other species, such as gold nanoparticles (Au-NPs) and streptavidin (SV). Vesel *et al*. demonstrated that the plasma can be used to immobilize streptavidin on the surface of polymer [18].

In this work, we utilize glow discharge plasma (GDP) [19,20], a conventional non-thermal plasma with high electron temperature and low gas temperature, to alter the configuration and surface properties of streptavidin coated gold nanoparticles (Au-NP-SV) and explore further the influence of GDP on the Au-NP-SV binding with other biomolecules such as biotinylated antibody (bAb). In this way, the reaction could occur near room temperature, so that the native structures of the biomolecules could be maintained. Atomic Force Microscope (AFM) was used to assess the morphology of the Au-NP biomolecular conjugates with SV and bAb before and after GDP treatment. We have found that the aggregation of Au-NP-SV conjugates increased significantly after GDP treatment compared to the control experiments.

## 2. Results and Discussion

**Figure 1:**
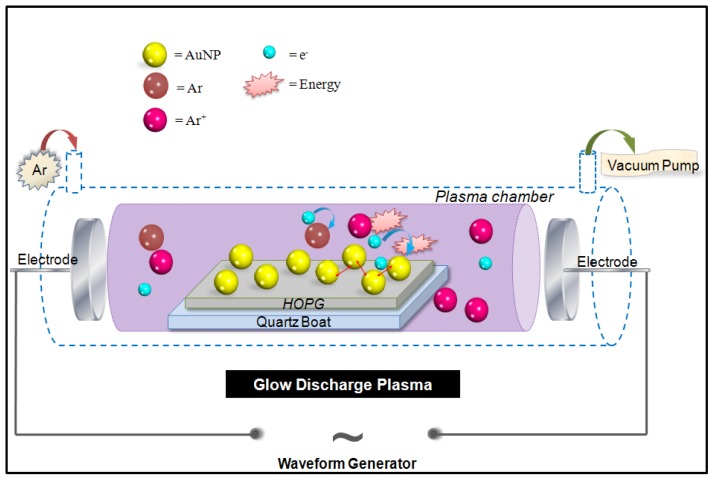
A schematic diagram showing the “glow discharge plasma” (GDP) generator. Argon (Ar, brown) upon high voltage generation between two electrodes will yield to the formation of argon ions (Ar^+^, purple) and electrons (e^−^, light blue). Gold nanoparticles Au-NPs (yellow) are deposited on top of the highly oriented pyrolytic graphite (HOPG) which is placed on top of a quartz plate.

Figure 1 shows a schematic diagram illustrating the working principle of glow discharge plasma (GDP). The main working principle of GDP is the generation of high voltage between two electrodes inside an evacuated glass chamber which will accordingly convert the gas used; argon gas (Ar, brown) for example, to ionic gas species (Ar^+^, purple) and electrons (e^−^, light blue) as illustrated in Figure 1.

By placing the adsorbed sample, for example Au-NPs (yellow) conjugated with SV and bAb, on top of an HOPG substrate which is then placed on top of a quartz plate inside the GDP evacuated glass tube, the generated Ar^+^ is expected to affect the adsorbed sample placed inside the chamber.

Plasma technology is known to influence the surface properties of surfaces and adsorbates [21]. It can change the properties of HOPG surface, for example, from hydrophobic to hydrophilic by using different sources of plasma such as argon or oxygen gases (see supporting information Figure S.1). Figure S.1 shows a schematic illustration of the influence of GDP on altering the nature of the HOPG surface from hydrophobic (Figure S.1A,B) to a hydrophilic (Figure S.1C). The influence is clearly illustrated by drop casting a drop of water on top of an untreated HOPG surface (hydrophobic), such that the water is located in one central place on top of the HOPG surface (*i.e.*, no complete surface wetting). Then, this HOPG with the adsorbed water droplet is placed inside the GDP chamber. One can clearly see that the water droplet completely spreads over the HOPG surface which indicates that the HOPG surface has become hydrophilic (*i.e.*, complete surface wetting).

Figure S.2 represents a Transmission Electron Microscope (TEM) image of citrate coated Au-NPs (Figure S.2A) and their predominant average size distribution (Figure S.2B) which is approximately 13 ± 1nm.

Plasma has also been used to influence the formation of metal nanowires inside inorganic matrices such as mesoporous silicates [18]. We believe that the use of plasma may affect the self-assembly, morphology, and binding capabilities of Au-NPs conjugated with SV and bAb.

### 2.1. GDP Treatment of Au-NP-SV and Its *in Situ* Mixing with Biotinylated Antibody (bAb)

**Figure 2:**
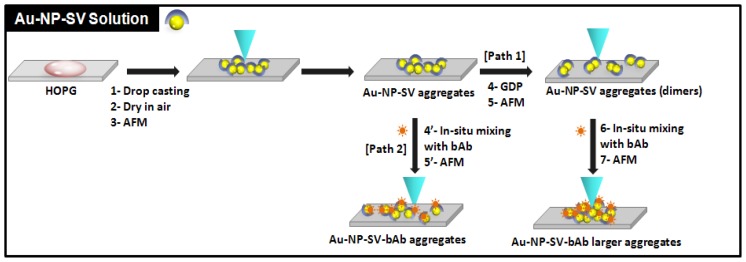
A schematic diagram illustrating the general layout of the consecutive steps of the experiments. Streptavidin coated gold nanoparticles (Au-NP-SV) are first adsorbed on top of the HOPG surface by drop casting, then dried in air and prepared for Atomic Force Microscopy (AFM) imaging. After successful AFM imaging, the adsorbed sample is placed inside the GDP glass chamber. After GDP treatment, the Au-NP-SV sample is then taken again for AFM imaging (path 1) followed by *in situ* mixing with bAb, and AFM images are recorded for the Au-NP-SV-bAb mixture. The control experiment is performed after the first AFM imaging (path 2) and successful *in situ* mixing with bAb followed by AFM imaging.

Figure 2 illustrates the general layout of the consecutive steps of the experiments before and after treatment with GDP. First, Au-NP-SV were adsorbed on top of a freshly cleaved HOPG surface by drop casting from a freshly prepared solution, then the substrate with the adsorbed sample on top of it was left to dry in air and then taken for AFM imaging (Figure 2: steps 1, 2, and 3). After successful AFM imaging, the adsorbed sample was taken and directly placed inside the GDP glass chamber (Figure 2: path 1, step 4). After GDP treatment of the sample, the Au-NP-SV sample was then taken outside the GDP chamber for AFM imaging (Figure 2: path 1, step 5).

**Figure 3:**
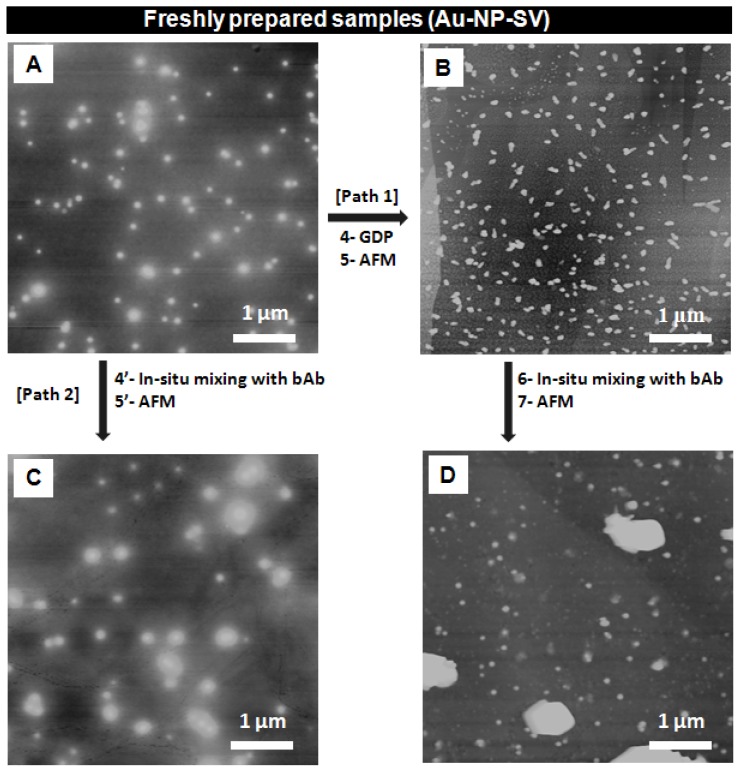
AFM images of freshly prepared Au-NP-SV adsorbed sample solutions on HOPG substrate before and after GDP treatment. (**A**) Before GDP, the Au-NP-SV conjugates appeared as bright features with a height profile ranging from 10 to 62 nm. *In situ* mixing of these structures with bAb (**C**) show structures with a height profile ranging from 10 to 48 nm (**C**, Path 2). After GDP of Au-NP-SV (**B**, path 1), binary structures appear in the images as “dimers” with a height profile ranging from 10 to 64 nm. *In situ* mixing of these dimeric features with bAb (**D**) show clusters with a height profile ranging from 10 to 267 nm. By comparing the mixed features in C and D, the height is clearly different.

After successful AFM imaging of the Au-NP-SV sample that was treated with GDP (Figure 3B), a drop of bAb was added (*in situ*) to the adsorbed Au-NP-SV/HOPG sample/substrate and AFM images were recorded for that *in situ* GDP treated mixture of Au-NP-SV-bAb (Figure 2: path 1, steps 6, 7 and Figure 3D). To check the influence of GDP on the binding affinity between Au-NP-SV and bAb, the non-GDP treated Au-NP-SV sample (control) on HOPG was imaged by AFM (Figure 2: path 2 and Figure 3A) and a drop of the bAb was added (*in situ*) to the control Au-NP-SV sample and AFM images were recorded for that *in situ* mixture of Au-NP-SV-bAb (Figure 2: path 2, steps 4′, 5′ respectively and Figure 3C).

**Figure 4:**
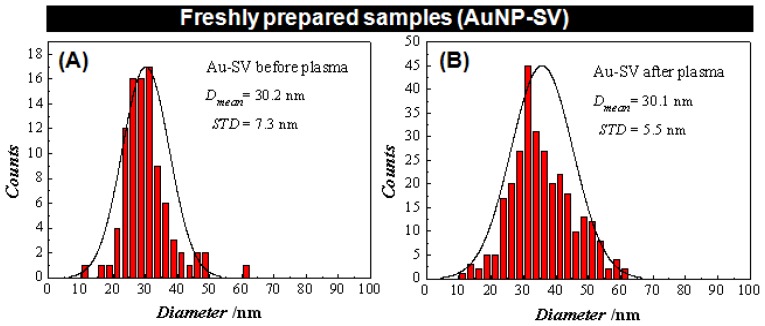

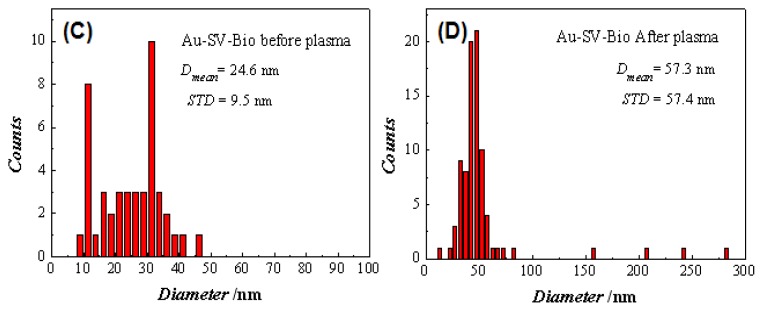
Histogram showing the height profiles of the AFM images represented in Figure 3 following the same experimental sequence.

Figure 3 shows the AFM images of freshly prepared Au-NP-SV samples before and after GDP treatment and also their *in situ* mixing with bAb as described in the general layout in Figure 2, paths 1 and 2, respectively. The corresponding height profile of the recorded AFM images in Figure 3 is shown in Figure 4.

Figure 3A shows an AFM image of the adsorbed sample solutions of Au-NP-SV conjugate structures before GDP, ranging from 10 up to 62 nm (as shown in Figure 4A). Figure 3B is an AFM image of the Au-NP-SV after treatment with GDP (path 1, steps 4 and 5) and shows the formation of binary structures “possibly dimers” with a height profile ranging from 10 up to 64 nm (as shown in Figure 4B). After *in situ* mixing of these Au-NP-SV dimeric structures with bAb (path 1, steps 6 and 7), Figure 3D shows larger features of Au-NP-SV-bAb with a height profile ranging from 10 up to 267 nm (as also shown in Figure 4D). Figure 3C shows the untreated Au-NP-SV sample (control) after *in situ* mixing with bAb (path 2, steps 4′ and 5′, respectively), with a height profile ranging from 10 up to 48 nm (as shown in Figure 4C).

By comparing the mixed structures shown in Figure 3C,D, and the values of their corresponding height profiles represented in Figure 4C,D, respectively, the following differences can be observed: (i) after GDP treatment, Au-NP-SV appears as “dimeric” structures with a slightly larger height profile (compared to the untreated Au-NP-SV), and (ii) *in situ* mixing of the GDP treated Au-NP-SV-bAb sample shown in Figure 3D appears as larger “clusters” with almost five times larger values of the height profile compared to the structures observed with the untreated Au-NP-SV-bAb (*in situ* mixed) in Figure 3C, respectively. It is not clear what the very large objects (almost 1 μm in diameter) in Panel 3D and their respective height profile in 4D are; they do not appear to be made up of clustered Au-NP alone since there is no granular detail near the edges of the objects. Moreover, these large chunks are what are counted here as clusters of increased size.

The above observations clearly demonstrate an apparent influence of GDP on the morphology and more importantly on the binding affinity of Au-NP-SV conjugates with similar conjugates as well as with bAb.

#### 2.1.1. The Influence of GDP on Au-NP-SV Binding

**Figure 5:**
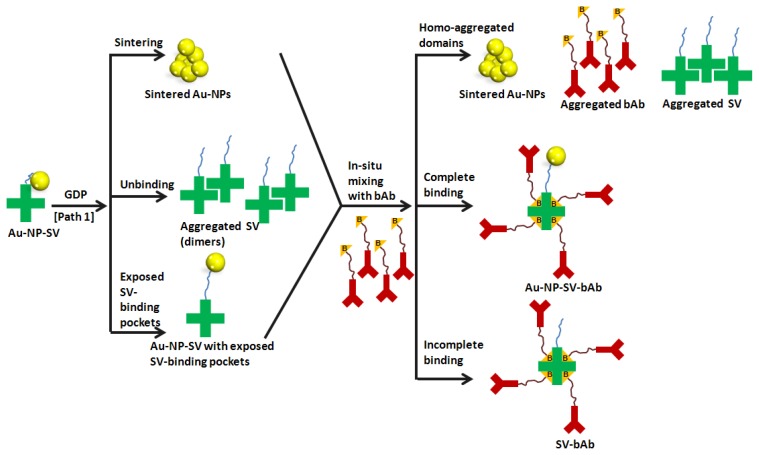
Schematic diagram illustrating the different possible mechanisms of the influence of GDP on the Au-NP-SV binding (left panel) and their *in situ* mixing with bAb (right panel). For display purposes, the Au-NP are represented by yellow spheres, SV units by green crosses attached to linkers in blue, and bAb by red Y-shape structures, respectively.

The small numbers of large chunks of material that appears following treatment with GDP are of unknown makeup. However, a number of different possible mechanisms shown in the schematic diagram in Figure 5 can be considered to assist in explaining the features observed in the recorded AFM images shown in Figure 3 and might shed light on the influence of GDP on the Au-NP-conjugates (discussed in this section) and on their *in situ* binding with bAb (will be discussed in the next section).

Three possible scenarios are illustrated in Figure 5 (left panel): (i) sintering of Au-NPs; (ii) unbinding of SV units from the Au-NP surface; and (iii) exposed SV pockets to bind bAb. It has been reported that sintering of nanoparticles can be carried out using a number of different external stimuli such as high temperature, pressure, chemical reagents, *etc.* [22–24]. A number of sintered nanoparticles have been reported such as silver, copper, iron, *etc.* [25–29]. In the case reported here, the high energy offered by the argon molecules and ions inside the plasma chamber can cause sintering of Au-NPs.

This can clearly be seen in Figure S.3A,B which shows AFM images of freshly prepared citrate coated Au-NP samples before and after GDP treatment and their corresponding height profiles. The AFM image of Au-NP in Figure S.3A before GDP treatment shows bright particles corresponding to individual and a few aggregated Au-NPs with a height distribution ranging from 8 nm to 33 nm (Figure S.3C). However, after GDP treatment of the Au-NP sample (Figure S.3B), the AFM image shows a number of larger structures with a height distribution ranging from 10 nm to 102 nm (Figure S.3D). This is a clear indication of an induced aggregation or sintering of Au-NPs upon treatment by GDP.

Another possible scenario is that upon exposure of the Au-NP-SV sample to GDP, the bonds between the SV units and the Au-NP were broken, leading to the unbinding of the SV units from the Au-NPs surface, and the separation of the SV units from the Au-NPs and their aggregation (dimerization) as appeared in Figure 3B. This will lead to sintering of the Au-NP on the surface, and also the aggregation (dimerization) of the SV units, leading to the formation of separated homo-aggregated domains. A possible explanation of the “dimeric” structures of the Au-NP-SV sample that appear after GDP treatment, as seen in Figure 3B, is the formation of intermolecular bonds between SV units (that have the ability to combine with each other during the GDP processing) leading to the formation of “dimeric” SV units.

The third possibility is related to the four pockets in the SV units that are suitable to bind four biotin molecules (bAb in this case). The ideal situation is that the SV units coat the Au-NPs without blocking their four pockets. However, one might expect that at least one of these pockets could be blocked by the Au-NP itself, thus decreasing the amount of bAb that can bind to one SV unit. In that respect, upon treating the Au-NP-SV sample with GDP, the high energy offered inside the plasma chamber might cause a conformational change around Au-NP-SV, leading to the exposure of some hidden SV pockets and thus to more binding with bAb (maximum four bAb units per one SV unit).

All these scenarios could occur at the same instance. However, due to the complexity of the observed structure and the limitation of the AFM image resolution, the exact scenario behind the formation of the features observed in Figure 3B and the nature of the exact bonds are not precisely known.

Figure S.4 shows Raman spectra of Au-NP-SV adsorbed on HOPG substrate before and after GDP treatment. It is clear that there is a shift in the Raman spectra after GDP treatment. This might be because the plasma assisted in aggregating (or sintering) Au-NP leading to the creation of larger Au-NP-conjugates out of the parent molecules. It could also cause freeing of some SV units (or subunits) that were originally bounded to the Au-NP surface that can form homo-clusters as described in Figure 5. Thus, upon GDP treatment, some of SV subunits might be exposed, allowing them to bind with more bAb, as explained above.

#### 2.1.2. The Influence of GDP on SV-bAb Binding

One of the most well-known examples appearing in the last several decades is the avidin-biotin system [30–34]. Based on molecular recognition, the system consists of a ligand, the small molecule biotin (vitamin H), and a receptor, the protein avidin that is present e.g., in egg white. The globular protein avidin is composed of four identical subunits, yielding four binding pockets that specifically recognize and bind to biotin, whereby each subunit binds one biotin molecule [35] as illustrated in Figure 5 (right panel). SV-biotin is one of the strongest binding biological entities [36]. The dissociation constant is of the order of 10^15^ M^−1^ and the bond, though not covalent, is found to be extremely stable, resisting harsh chemical conditions and elevated temperature [37].

Moreover, the high affinity of SV-biotin binding is related to: (1) the high shape complementarily between the protein binding pocket in SV units and the biotin, (2) the extensive hydrogen bonds formed in the SV-pockets with biotin, and finally (3) the SV-pocket itself is hydrophobic and there are many Van der Walls and hydrophobic interactions occurring with biotin when the latter is within the SV binding pocket [38].

As a general rule in bioconjugation of nanoparticles, proteins like streptavidin, bovine serum albumin, cytochrome C and others often interact with the negatively charged surface of Au-NPs through non-covalent electrostatic interactions between the positively charged amino acids side chains of the proteins and the negatively charged surface of the Au-NPs [37].

An important question concerning this point is raised: Is it possible to enhance/decrease this SV-biotin binding by using GDP? Our AFM results in Figure 3 show that some aggregation has occurred upon treatment of the Au-NP-SV sample with GDP followed by *in situ* mixing with bAb.

The corresponding increase in height profile shown in Figure 4 is very complicated to explain and to be completely understood, and therefore we will suggest several different scenarios illustrated in Figure 5 that could shed light to gain more insights into that point. Tryptophan and lysine residues in each SV subunit are involved in forming the binding pocket between SV and bAb, where hydrophobic, Van der Waals forces and hydrogen bonding are formed during the binding [35].

Taking into account the possible mechanisms illustrated in Section 2.1.1 on the influence of GDP on the Au-NP-conjugates, here we will shed light onto the following experimental step, which is the *in situ* mixing of the GDP treated Au-NP-SV samples with bAb.

Upon exposing the Au-NP-SV samples to GDP, three possible mechanisms are proposed which might all occur simultaneously or in part. By adding bAb to the GDP treated Au-NP-SV sample, three scenarios are proposed as shown in Figure 5 (right panel): (i) Homo-aggregated domains including separation of sintered Au-NPs or SV units or bAb units on the HOPG surface; (ii) Complete binding which is the ideal case where the Au-NP-SV unit binds four bAb units in SV-pockets; and (iii) Incomplete binding where Au-NP are detached from the SV unit, leaving it to be adsorbed on the HOPG surface, and the SV unit will then binds four bAb units in SV-pockets.

The formation of large structures, as observed in Figure 3D, with a height profile ranging from 10 to 267 nm might in this case be due to the formation of either homo-aggregated domains of clustered Au-NPs, SV units or bAb as suggested in our first mechanism or it could also be a combination of the three mechanisms that might occur simultaneously too.

Is it possible that the GDP enhanced the binding between SV and biotin so that one SV unit could bind more than four biotin molecules? The answer to this question is very complicated; especially if we consider that the SV units are bound to Au-NP, so they are not totally free but still have some ability to fold. It is also possible that there are some binding sites that are not accessible to biotin molecules as proposed in Figure 5 (left panel, the last mechanism).

It is clear from Figure 4D that the height profile increased by more than four times the value after GDP treatment followed by bAb addition. Also, as observed, the GDP treatment increased the hydophilicity of the sample surface (as can be seen on the bare HOPG surface and the adsorbed water droplet on top of it in Figure S.1), so, a possible explanation for the clusters formed after GDP treatment could also be due to an increased number of hydrogen bonds available for biotin-SV binding leading to binding of more bAb molecules. It is also known that SV is highly stable and keeps its native structure under harsh conditions, such as under a wide range of pH values, different buffer conditions and different chemical modifications [35]. This means that GDP treatment, according to the obtained results, could affect the native protein structure or increase the number of bAb bonded to SV units. However, the second explanation seems very implausible. It might be more likely that the GDP affects the residual charge on the gold particles rather than exposing new biotin binding sites on the SV. The experimental data presented here provide a number of plausible mechanisms for the clustering, however, one cannot certainly know which scenario is most plausible, especially with quite a number of parameters taking part (such as different bonds, number of constituents, homo- and hetero-aggregation, *etc.*) and all the different possibilities and pathways that might occur simultaneously.

## 3. Experimental Section

### 3.1. Gold Nanoparticles-Streptavidin Conjugates (Au-NP-SV)

Streptavidin coated gold nanoparticles (Au-NP-SV) was purchased from BioAssay Works (Ijamsville, MD, USA). The biotinylated antibody (bAb) mouse monoclonal anti-Prostate specific antigen (PSA) was purchased from HyTest Ltd., Turku, Finland. Deionized water was used for preparation of citrate Au-NPs and Au-NP-SV solutions. The concentration of the prepared solutions of Au-NP-SV was 3.8 mM (15 OD at lambda max of 533 nm), while the Au-NP-SV-bAb used was 50 μg.

### 3.2. Glow Discharge Plasma (GDP)

The glow discharge plasma setup was similar to that described previously [39,40]. Briefly, the sample was placed in the glow discharge chamber, which was a quartz tube (i.d. 35 mm) with two stainless steel electrodes (o.d. 30 mm) and a mechanical vacuum pump. When the pressure was adjusted to 100–200 Pa, the glow discharge plasma was generated by applying a voltage of 900 V to the electrode, using a high-voltage amplifier (Trek, 20/20B), with argon (99.999%) as the plasma-forming gas. The signal input for the high-voltage amplifier was supplied by a function/arbitrary waveform generator (Hewlett-Packard, 33120A) with a 100 Hz square wave. The current was 4.5 mA. The gas temperature of plasma was measured by infrared imaging (Ircon, 100PHT), indicating that the reaction was conducted at ambient temperature. The discharge time for each sample was only 2 min.

### 3.3. Sample Preparation

Au-NP-SV solution (20 μL) was deposited onto a freshly cleaved highly oriented pyrolytic graphite (HOPG, grade ZYB from NT-MDT Co., Moscow, Russia) and treated at room temperature in an argon glow discharge system. After AFM imaging, 10 μL of the biotinylated antibody (bAb) mouse monoclonal anti-Prostate specific antigen (PSA) was deposited on top of the adsorbed Au-NP-SV/HOPG, and was left to dry in air for 30 min.

### 3.4. Atomic Force Microscopy (AFM)

The sample surface was scanned in air under ambient conditions in a tapping mode (TM-AFM) using a MultiMode AFM with a Nanoscope V controller (Veeco Instruments, Santa Barbara, CA, USA) with 40 N/m force constant cantilever (RTESP, Veeco Instruments, Santa Barbara, CA, USA), at a typical resonance frequency υ_0_ ≈ 290 kHz, a spring constant of 40 N·m^−1^ and a normal tip radius of approximately 10 nm, and a vertical engagement (JV) 125 μm scanner. After engagement, the tapping amplitude set point was typically 0.4 V and the scan rates ranged from 0.8 to 1.5 Hz. During the AFM imaging, the highest resolution was in most cases obtained with minimal loading forces applied and optimized feedback parameters. Several AFM images, all 512 × 512 pixels, were obtained from separate locations across the HOPG surfaces to ensure reproducibility of the results. All the AFM images were analyzed using a Nanoscope software version 7.30 (Veeco Instruments: Santa Barbara, CA, USA, 2008). In the histograms in Figure 4, the individual events have been counted one by one in each of the given images shown in Figure 3, including all of the particles (either big or small). Each of the images has been flattened in 0th or 1st order and the Section tool available in Veeco Nanoscope software version 7.30 has been used to determine the maximum vertical distance (depth) for each particle.

## 4. Conclusions

The experimental strategy employed in this study represents a new approach in which the self-assembly, morphology, binding affinity and aggregation of bio-nanomaterials can be directly affected and measured after exposure to GDP as an external stimulus. The analysis of the AFM images of Au-NP-SV and the *in situ* mixed samples of Au-NP-SV with bAb showed a considerable change in the aggregation and morphology of these Au-NP-conjugates after treatment with GDP compared to the untreated samples. Different plausible scenarios explaining the mechanisms upon exposure to GDP have been proposed. These results show that by using an external stimulus such as GDP, it is possible to introduce a different pathway through which the Au-NP-SV molecules can bind together and with bAb, leading to the formation of a variety of new supramolecular nanostructures.

The experimental protocol discussed above can be generalized for other nanomaterials and surfaces by using other chemically functionalized (or coated) nanoparticles, using different surfaces (or chemically modified surfaces), or different sources of plasma (Ar, O, *etc.*) (work in progress) which will result in a wealth of information that is needed to control the surface properties of adsorbed nanomaterials and nanostructured surfaces. Moreover, theoretical studies will be very useful in depicting the most favourable mechanism taking place at the molecular level of adsorbed samples before and after exposure to GDP.
